# Photocatalytic Activities Enhanced by Au-Plasmonic Nanoparticles on TiO_2_ Nanotube Photoelectrode Coated with MoO_3_

**DOI:** 10.1186/s11671-017-2327-y

**Published:** 2017-10-06

**Authors:** Chia-Jui Li, Chuan-Ming Tseng, Sz-Nian Lai, Chin-Ru Yang, Wei-Hsuan Hung

**Affiliations:** 1 Department of Material Science and Engineering, Feng Chia University, Taichung, Taiwan; 2 Department of Materials Engineering, Ming Chi University of Technology, New Taipei City, Taiwan

**Keywords:** Metal oxide, Core–shell structure, Plasmonic nanoparticles, Photocatalysis reaction

## Abstract

Although TiO_2_ was formerly a common material for photocatalysis reactions, its wide band gap (3.2 eV) results in absorbing only ultraviolet light, which accounts for merely 4% of total sunlight. Modifying TiO_2_ has become a focus of photocatalysis reaction research, and combining two metal oxide semiconductors is the most common method in the photocatalytic enhancement process. When MoO_3_ and TiO_2_ come into contact to form a heterogeneous interface, the photogenerated holes excited from the valence band of MoO_3_ should be transferred to the valence band of TiO_2_ to effectively reduce the charge recombination of photogenerated electron–hole pairs. This can efficiently separate the pairs and promote photocatalysis efficiency. In addition, photocurrent enhancement is attributed to the strong near-field and light-scattering effects from plasmonic Ag nanoparticles. In this work, we fabricated MoO_3_-coated TiO_2_ nanotube heterostructures with a 3D hierarchical configuration through two-step anodic oxidation and a facile hydrothermal method. This 3D hierarchical structure consists of a TiO_2_ nanotube core and a MoO_3_ shell (referred to as TNTs@MoO_3_), as characterized by field emission scanning electron microscopy and X-ray photoelectron spectroscopy.

**Electronic supplementary material:**

The online version of this article (10.1186/s11671-017-2327-y) contains supplementary material, which is available to authorized users.

## Background

Rapid technological development has been accompanied by an increased demand for energy. Consequently, research into alternative energy sources has become popular over the past decade, with many scientists focused on renewable energy sources with low carbon emissions and minimal environmental impact. These include solar energy [1, 2], geothermal heat [3, 4], tides [5], and various forms of biomass [6, 7]. Photocatalytic water splitting, as the most direct method for achieving the goal of clean and renewable energy [8], is also the most investigated method of directly converting solar energy into chemical energy. Some common means of promoting energy conversion efficiency include increasing the reaction area, catalyst deposition, and compositing with secondary materials; for example, synthesizing specific microstructures [9–11], depositing Pt as a catalyst [12, 13], and combining two different metal oxides [14–16].

TiO_2_ nanotube (TNT) arrays have received considerable attention for their large surface area, robust photocatalytic activity, and vectorial charge transfer properties [17–19]. However, the practical application of TiO_2_ is restricted by its wide band gap (3.2 eV). This results in absorbing only UV light, which accounts for 4% of total sunlight, greatly limiting its photocatalytic activity in the visible light region. In addition, the high recombination rate of TiO_2_ lowers the efficiency of photocatalytic activity. To solve these problems, many studies have focused on extending the absorption edge of TiO_2_ into the visible light region, including doping with nitrogen or other nonmetals [20, 21], surface modification with noble metals [22, 23], and coupling with narrow-band-gap semiconductors [14–16].

Molybdenum trioxide (MoO_3_) is a p-type metal oxide semiconductor with a high work function and excellent hole conductivity; therefore, it is widely used in organic solar cells and organic light-emitting diodes [24, 25]. MoO_3_ has a band gap of approximately 2.8 eV, with 20–30% ionic character and the capacity to absorb both UV and visible light [26]. The valence and conduction band positions of MoO_3_ are both lower than those of TiO_2_. Hence, a heterojunction between TiO_2_ and MoO_3_ might enhance photocatalytic activity by decreasing the charge recombination and promoting the charge transfer process [27]. Under visible light irradiation, the holes excited from the valence band of MoO_3_ should be transferred to the valence band of TiO_2_, to reduce the charge recombination of photogenerated electron–hole pairs.

Plasmonic photocatalysis has recently facilitated the rapid enhancement of photocatalytic efficiency under visible light irradiation [28, 29]. A surface plasmon is a surface electromagnetic wave on the metal–dielectric interface, widely used in optical, chemical, and biological sensing for the high sensitivity of its resonant waves. The surface plasmon resonance effect is confined to the metal surface to form a highly enhanced electric field [30]. When the particular resonance frequency of plasmonic metal nanoparticles matches that of the incident photon, strong electric field forms near the surface of the metal. Furthermore, tunable interactions between incident visible light and excited plasmonic nanoparticles are achieved by controlling their sizes and shapes, as well as the dielectric constant of the surrounding environment [31–33].

**Scheme 1:**
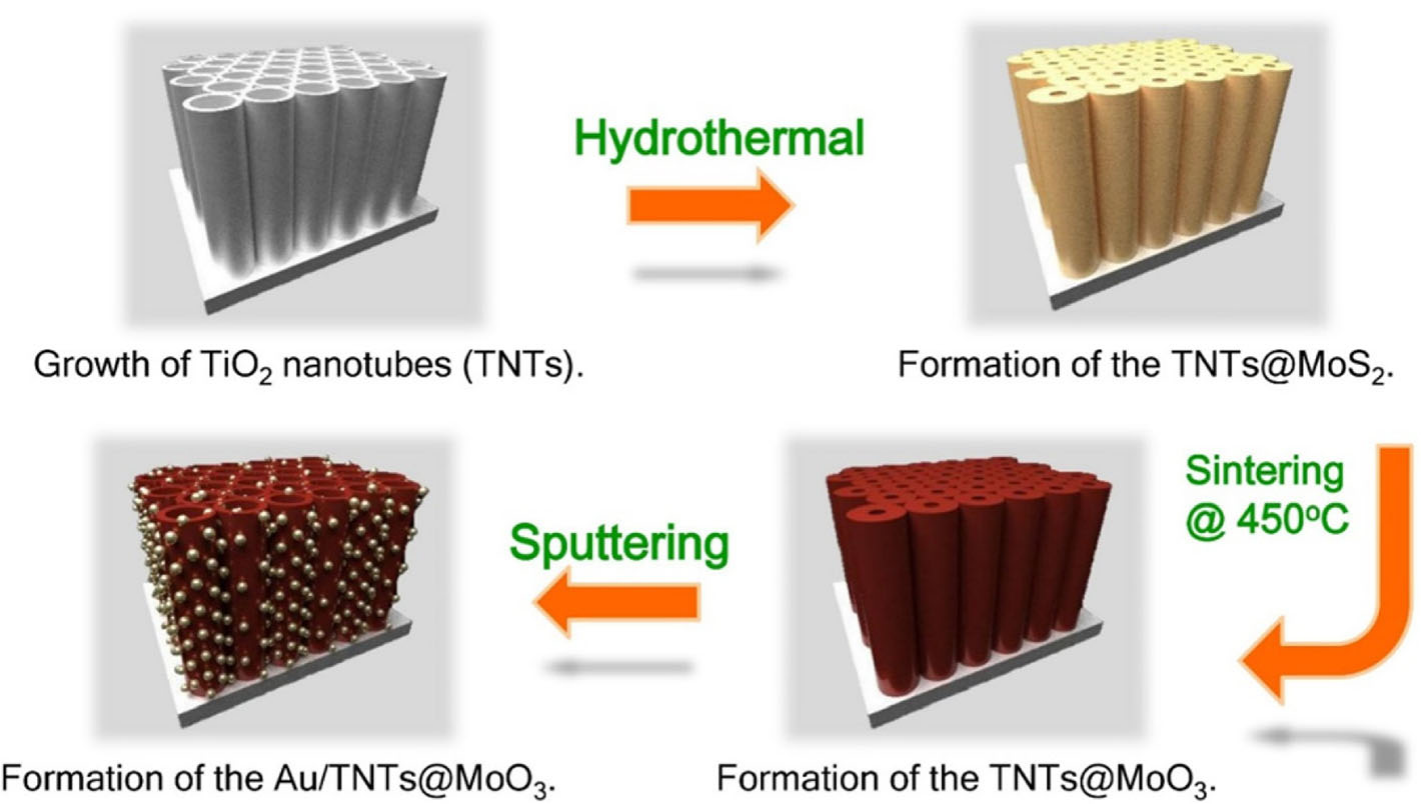
Charge separation at the interface of the TiO_2_–MoO_3_ composite

In the present work, we first synthesized MoS_2_ coating on the surface of TNTs through a hydrothermal method. MoS_2_ was then oxidized to MoO_3_ through a simple annealing process (Scheme 1). This process enabled high coverage of MoO_3_ nanoscale particles with a highly ordered structure. To further enhance the photocatalytic water-splitting performance, we introduced a surface plasmon resonance (SPR) effect.

## Methods

### Fabrication of the TiO_2_ Nanotubes

The TNTs were fabricated by a two-step anodic oxidation method. Prior to the anodic oxidation process, the titanium foil was cut to size and placed in acetone, then ethanol, then deionized (DI) water, and then subjected to ultrasonic vibration for 5 min. Anodic oxidation was carried out using a conventional two-electrode system with the Ti foil as an anode and a carbon rod as a cathode. All electrolytes consisted of 0.3 wt% ammonium fluoride (NH_4_F) in ethylene glycol (C_2_H_6_O_2_, EG) solution with 5 vol% water. All processes were carried out at room temperature.

In the first step of anodic oxidation, the Ti foil was anodized at 60 V for 30 min; the as-grown nanotubes were subsequently removed in 1 M HCl by ultrasonic vibration. The same Ti foil then underwent a second anodic oxidation process at 60 V for 30 min. After both steps were completed, the prepared TNTs were washed with ethanol and DI water. The TNTs were annealed in air at 450 °C for 4 h at a heating rate of 2 °C/min to form the anatase TNTs.

### Synthesis of TNTs@MoO_3_ Core–Shell Structure

The TNTs@MoO_3_ core–shell structure was synthesized with a hydrothermal method and a simple annealing process. MoS_2_ nanosheets were synthesized by the following procedures: 0.12 g of sodium molybdate (Na_2_MoO_4_·2H_2_O) and 0.24 g of thioacetamide (TAA) were dissolved in 80 mL of DI water under vigorous stirring for 15 min. Subsequently, the transparent solution and as-grown TNTs were transferred into a 100-mL Teflon-lined stainless steel autoclave, which was sealed and heated to 200 °C at a heating rate of 3 °C/min and held for 24 h. After the autoclave was cooled to room temperature, the prepared TNTs@MoS_2_ were washed with DI water. The TNTs@MoS_2_ were annealed in air at 450 °C for 4 h with a heating rate of 2 °C/min to form the TNTs@MoO_3_ core–shell structure.

### Deposition of Au Nanoparticles

The plasmonic cocatalyst photoelectrodes (Au/TNTs@MoO_3_) were fabricated with the prepared TNTs@MoO_3_ cocatalytic core–shell structure through the hydrothermal method, followed by the standard sputtering deposition of Au nanoparticles.

### Characteristic Analysis and Photocurrent Measurements

The microstructures and morphologies of the samples were examined using field emission scanning electron microscopy (FE-SEM) and energy-dispersive X-ray spectroscopy (EDS). To confirm the bonding energy of the developed TiO_2_, MoS_2_, and MoO_3_ photoelectrodes, X-ray photoelectron spectroscopy (XPS) was employed. Finally, the photocatalytic reaction was measured in 1 M NaOH solution by operating three terminal potentiostats at room temperature under 532-nm laser irradiation with a 1-mm diameter spot size.

## Results and Discussion

**Fig. 1:**
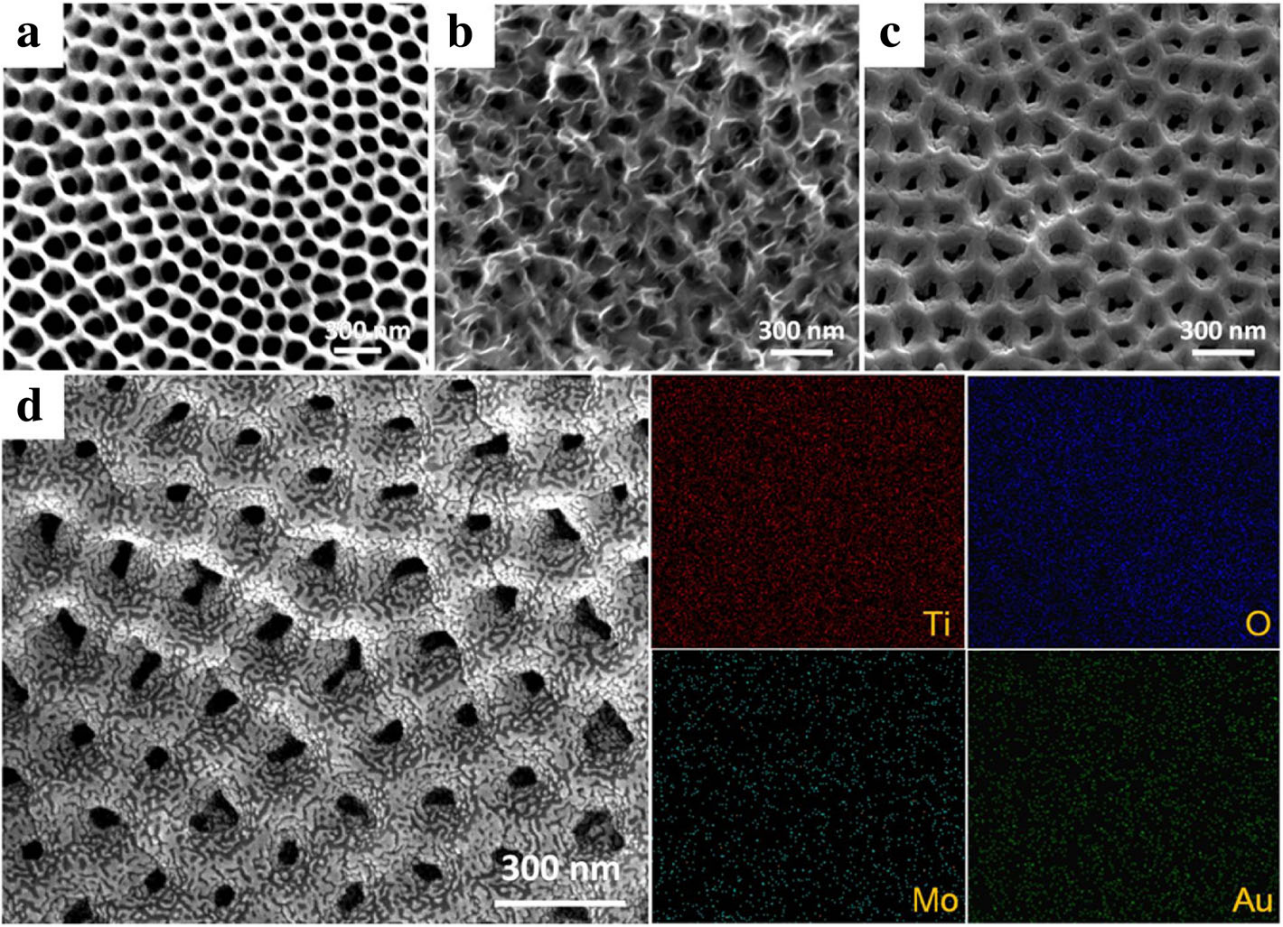
SEM images of **a** TNTs, **b** TNTs@MoS_2_, **c** TNTs@MoO_3_, and **d** Au/TNTs@MoO_3_ (left), as well as EDS mapping (right)

Figure 1 shows the SEM images and EDS mapping of the prepared samples. Figure 1a–c shows the SEM images of the TNTs, TNTs@MoS_2,_ and TNTs@MoO_3_. The SEM image of TNTs obtained by two-step anodic oxidation of Ti foil in 0.3 wt% NH_4_F contained in ethylene glycol solution (Fig. 1a) exhibited uniform pore size (100–120 nm). After the core–shell structure was formed with MoS_2_ covered through the hydrothermal method, the porous structure of TNTs was not blocked to reduce the active reaction sites (Fig. 1b). Subsequently, the TNTs@MoO_3_ core–shell structure was formed by a simple annealing process in the tube furnace (Fig. 1c). Figure 1d shows the SEM image and EDS mapping of Au/TNTs@MoO_3_, providing clear information about the Ti, O, Mo, and Au. The uniform deposition of the island-like Au nanoparticles, observable on top of the TNTs@MoO_3_, facilitated the generation of the SPR effect.

**Fig. 2:**
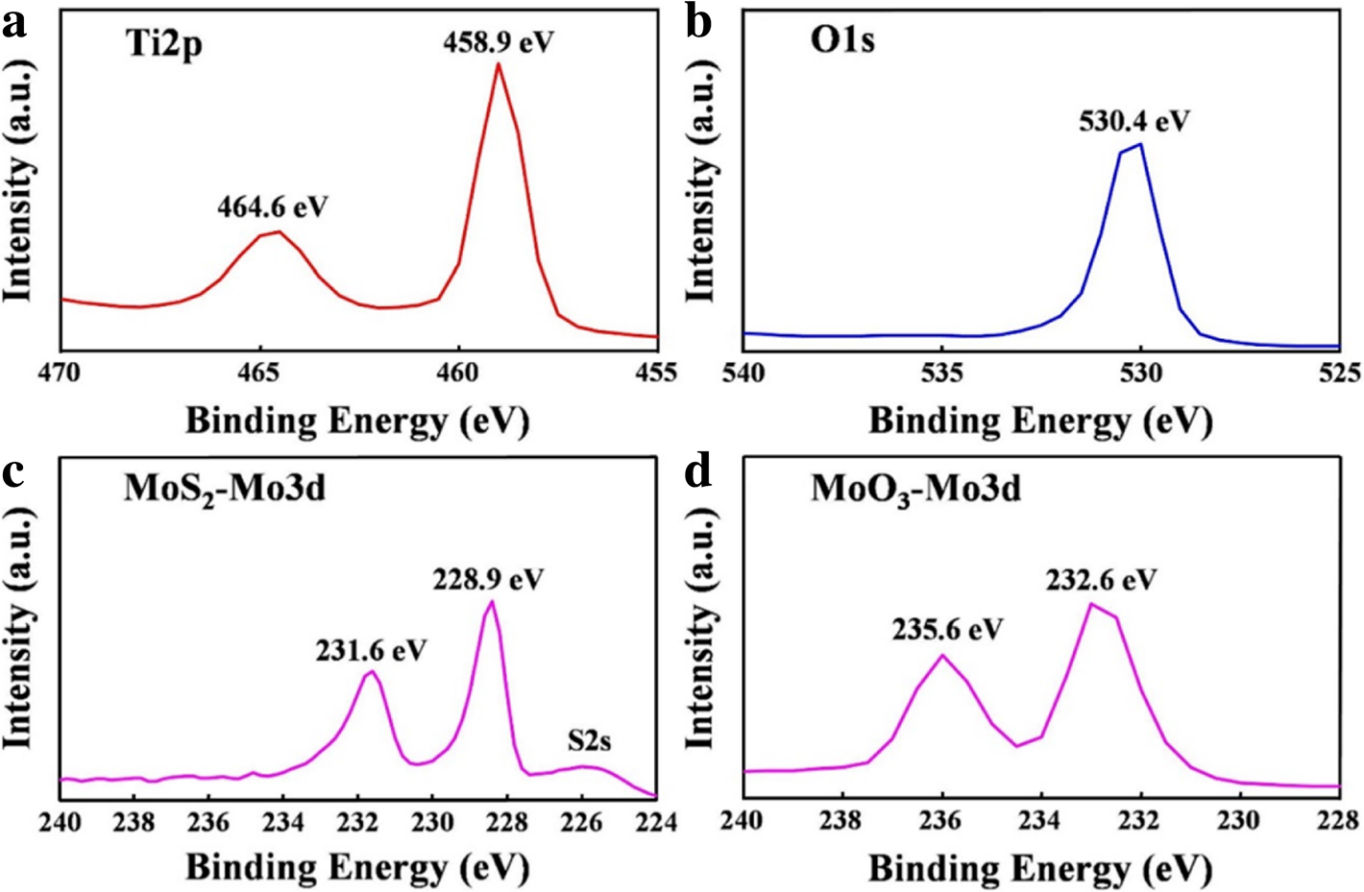
XPS analysis of **a** Ti2p, **b** O1s, **c** Mo3d of MoS_2_, and **d** Mo3d of MoO_3_

XPS was used to investigate the chemical states of the TNTs@MoO_3_ after conversion from TNTs@MoS_2_ through a simple annealing process (Fig. 2). Three characteristic peaks of Ti and O can be observed in Fig. 2a, b. The binding energies at the Ti2p1, Ti2p3, and O1s peaks are 464.6, 458.9, and 530.4 eV, respectively. In Fig. 2c, a Mo3d3 peak at 231.6 eV and Mo3d5 peak at 228.9 eV can be identified, indicating the chemical composition of MoS_2_ in the TNTs@MoS_2_. In addition, a weak peak appearing at approximately 226 eV is the signal peak of S2s. The Mo3d3 and Mo3d5 peaks in Fig. 2d with binding energies of 235.6 and 232.6 eV are ascribed to Mo^6+^ in MoO_3_. Therefore, the XPS investigations confirm that the red shift of the spectrum reflects the conversion of the Mo element valence from tetravalent to hexavalent.

**Fig. 3:**
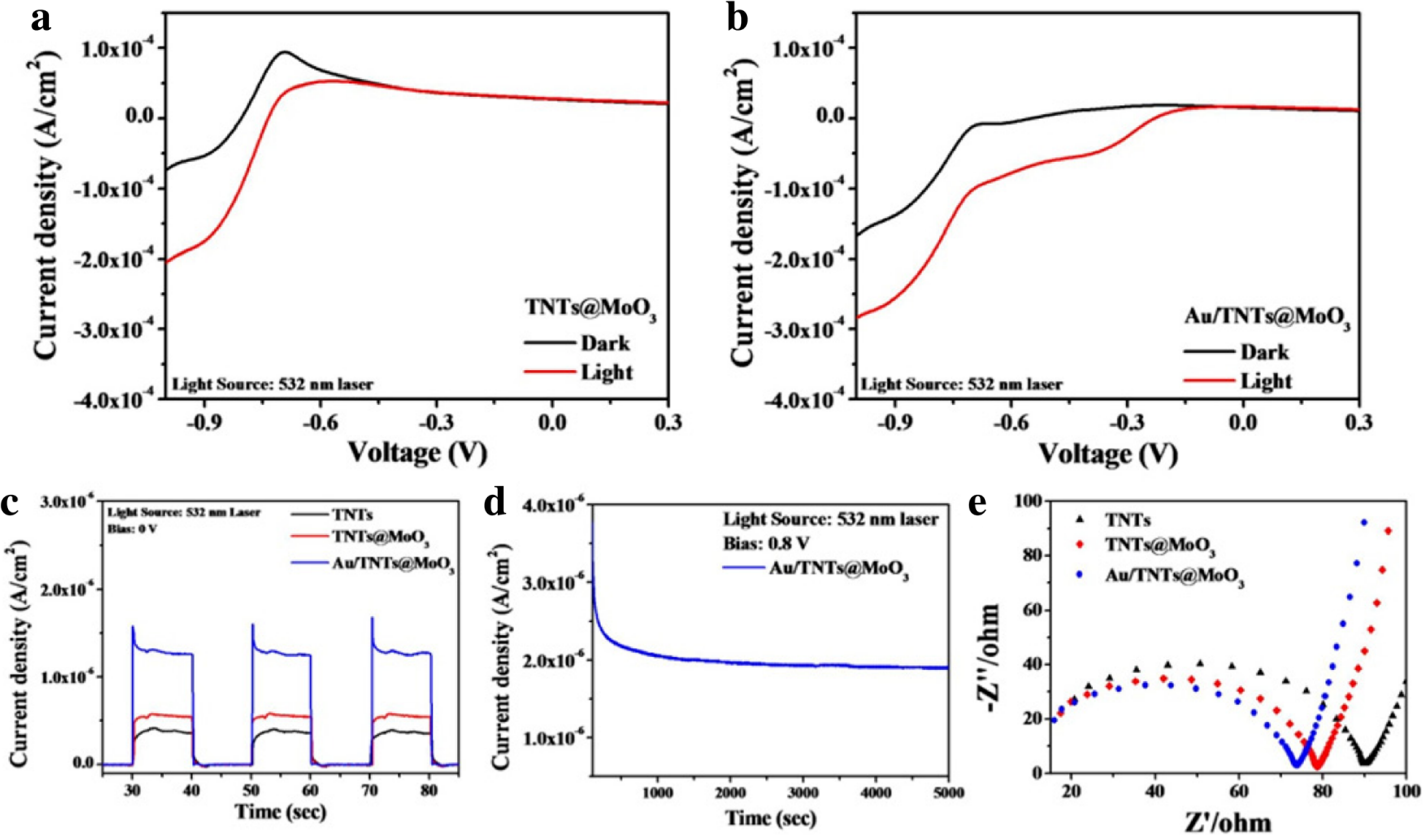
Linear sweep curves of photoelectrodes **a** without and **b** with light irradiation, and photocurrent responses at **c** 0 V (light source: 532-nm laser). **d** Prolonged photocurrent measurements under 532-nm laser irradiation. **e** Nyquist plots of various photoelectrodes

The photocatalytic water-splitting performance of the prepared photoelectrodes was measured under 532-nm laser irradiation. Figure 3a, b shows the photocurrent response (I–V curves) of TNTs@MoO_3_ and Au/TNTs@MoO_3_. According to the results, TiO_2_@MoO_3_ exhibits a higher photocurrent because of the enhanced charge separation rate at the TiO_2_@MoO_3_ heterogeneous interface (shown in Fig. 3a). Furthermore, with the integration of Au nanoparticles, Au/TNTs@MoO_3_ presented a photocurrent response approximately 1.5 times higher than TNTs@MoO_3_ at the bias voltage of −1 V. Figure 3c shows the I–T curves of the TNTs, TNTs@MoO_3_, and Au/TNTs@MoO_3_ at the bias voltage of 0 V. As shown in Fig. 3c, the photocurrent response was higher again in the Au/TNTs@MoO_3_ structure compared to the TNTs@MoO_3_ photoelectrode without the application of bias voltage. The photocurrent response of Au/TNTs@MoO_3_ could be enhanced through the simple SPR effect.

To further investigate the photocatalytic activity of the prepared photoelectrodes, we also examined the extended photocurrent responses and electrochemical impedance spectroscopy to understand the photocurrent stability and the charge transfer at the photoelectrode–electrolyte interfaces (Fig. 3d, e). The extended stability of the photoelectrode with the optimal performance, Au/TNTs@MoO_3_, was examined under 532-nm laser irradiation for approximately 1.5 h (Fig. 3d). At the applied voltage of 0.8 V, the photocurrent remained at 57% of its initial value. Figure 3e shows the Nyquist plots of all three tested photoelectrodes under 532-nm laser irradiation recorded at a DC potential of 1.23 V versus RHE and an AC potential frequency range of 10^6^–1 Hz with an amplitude of 1 V under 532-nm laser irradiation. According to the results, smaller semicircle diameters can be observed in the Au/TNTs@MoO_3_ sample, indicating a lower transport impedance for charge carriers. The formation of a heterogeneous interface between TiO_2_ and MoO_3_ is confirmed to facilitate charge transfer and enhance photocatalytic activity through the excellent carrier conduction properties of the Au nanoparticles.

## Conclusions

### Supporting information

In the supporting information (Additional file 1) we performed the Raman spectra analysis of MoS_2_ layer, the related thickness and average pore size of SEM images of TNTs, and the enhancement mechanism of the system.

In this study, we successfully fabricated a TNTs@MoS_2_ core–shell heterostructure by a two-step anodic oxidation process and a facile hydrothermal method to form a TNTs@MoO_3_ core–shell structure through a simple annealing process. According to the results, a MoO_3_ coating on a photoelectrode can enhance its utilization of photons in the visible region. Moreover, with the integration of plasmonic Au nanoparticles, a significant improvement in the water-splitting photocurrent was observed compared to pure TiO_2_ nanotubes under visible light irradiation. The energy band engineering of the TNTs@MoO_3_ heterostructure favors charge transfer and suppresses photogenerated electron–hole pair recombination between MoO_3_ and TiO_2_, leading to enhanced photocatalytic activity.

## Additional file

Additional file 1: Supporting information. (DOCX 1074 kb)
