# Supplementary material for: Photocatalytic Activities Enhanced by Au-Plasmonic Nanoparticles on TiO2 Nanotube Photoelectrode Coated with MoO3
Source: Nanoscale Res Lett. 2017 Oct 6;12:560. doi: 10.1186/s11671-017-2327-y (PMC5630543; doi:10.1186/s11671-017-2327-y)
Supplement: Additional file 1: — Supporting information. (DOCX 1074 kb) [file 11671_2017_2327_MOESM1_ESM.docx]

**Supporting information**

**Photocatalytic Activities Enhanced by Au-Plasmonic Nanoparticles on TiO_2_ Nanotube Photoelectrode Coated with MoO_3_**

Chia-Jui Li^1^, Chuan-Ming Tseng^2*^, Sz-Nian Lai^1^, Chin-Ru Yang^1^, and Wei-Hsuan Hung^1*^

^1^Department of Material Science and Engineering, Feng Chia University, Taichung, Taiwan

^2^Department of Materials Engineering, Ming Chi University of Technology, New Taipei City, Taiwan

*E-mail: [whung@fcu.edu.tw](file:///D:\Xioufei\Lab\Paper%20Submit\2017_Paper%20Submit\李佳叡\20170807_Nanoscale%20Research%20Letters\whung@fcu.edu.tw) (Wei-Hsuan Hung), [cmtseng@mail.mcut.edu.tw](file:///D:\Xioufei\Lab\Paper%20Submit\2017_Paper%20Submit\李佳叡\20170807_Nanoscale%20Research%20Letters\cmtseng@mail.mcut.edu.tw) (Chuan-Ming Tseng)

**
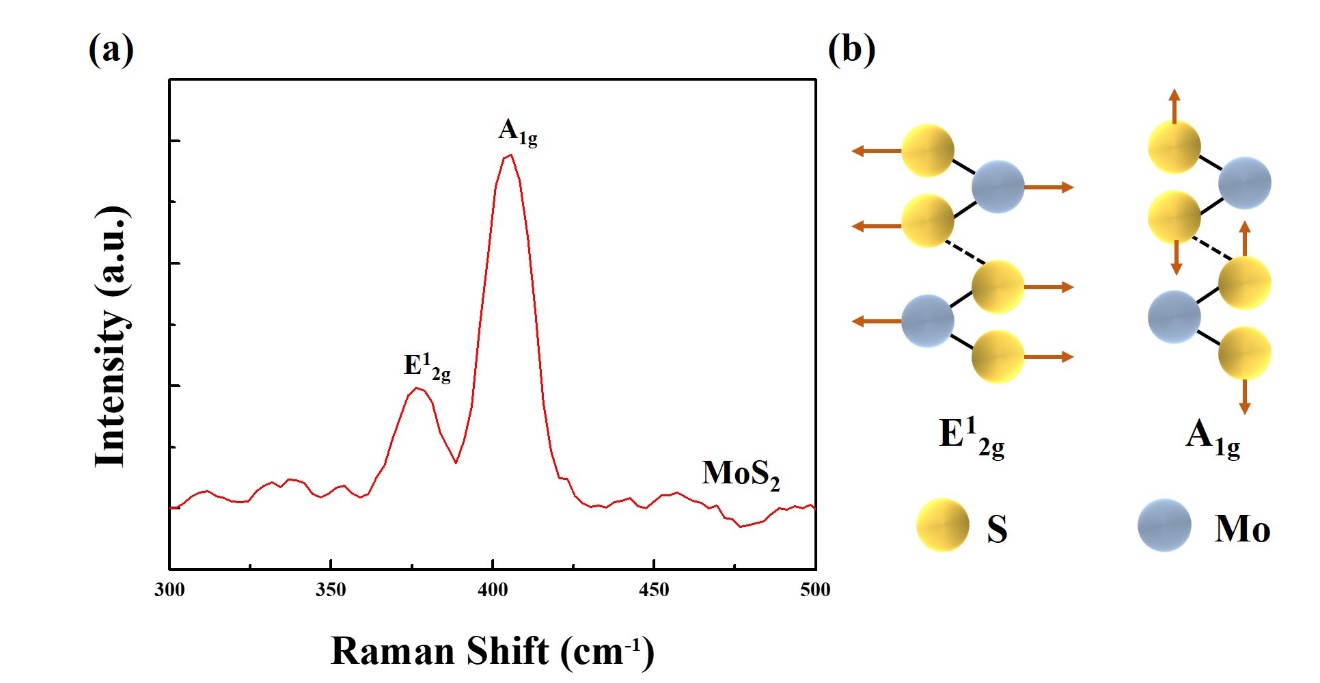
**

**Figure S1.** (a) The Raman spectra of MoS_2_ layer. (b) The schematic representation of two domain modes of MoS_2_

The Raman spectra of MoS_2_ is shown in Figure S1(a), the vibration of A_1g_ peak is 405.8 cm^-1^, and the E^1^_2g_ peak is 376.3 cm^-1^, which is similar to other studies with some red-shifted.[^1-3^](#_ENREF_1) In general, the frequency difference between E^1^_2g_ and A_1g_ peak is usually used to determine the layer number of MoS_2_ crystal.[^2^](#_ENREF_2)^,^ [^4^](#_ENREF_4) The frequency difference of the MoS_2_ here is 29.5 cm^-1^, which is consistent with bulk MoS_2_.[^4^](#_ENREF_4) In order to make the photocatalyst achieve another enhancement, we transform the formation of MoS_2_ to MoO_3_ instead, according to their excellent coating performance. Figure S1(b) exhibits the schematic representing of two domain modes of MoS_2_: E^1^_2g_ and A_1g_, where E^1^_2g_ represents the in-plane vibration mode of Mo and sulfur atoms while A_1g_ represents the out-of-plane vibration mode of sulfur atoms.[^5^](#_ENREF_5)

**
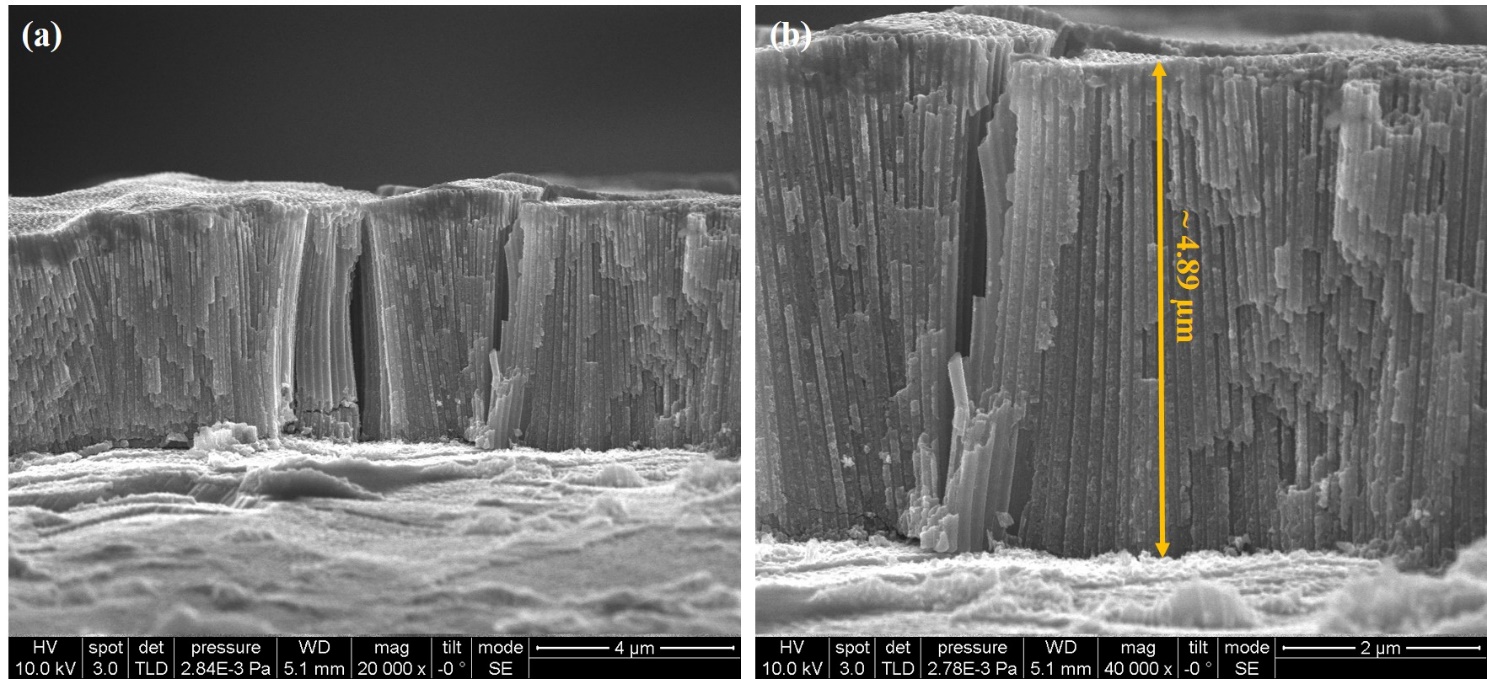
**

**Figure S2.** SEM cross section of TNTs. (a) Low magnification. (b) High magnification.

The related thickness of TNTs are shown in Figure S2. It is 4.89 µm approximate in average.


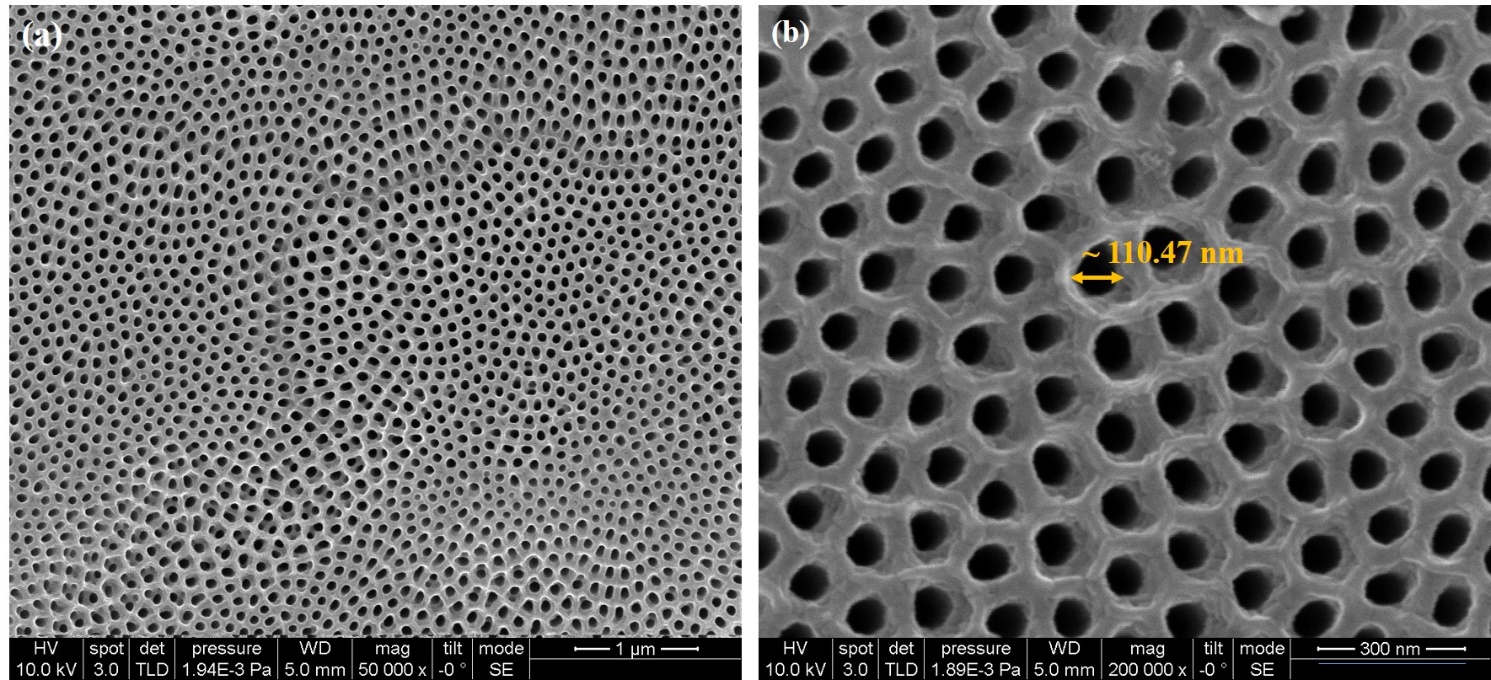


**Figure S3.** The SEM images of TNTs. (a) Low magnification. (b) High magnification.

The related thickness of TNTs are shown in Figure S2. It is 4.89 µm approximate in average. And the average pore size is 110.47 nm shown in Figure S3.


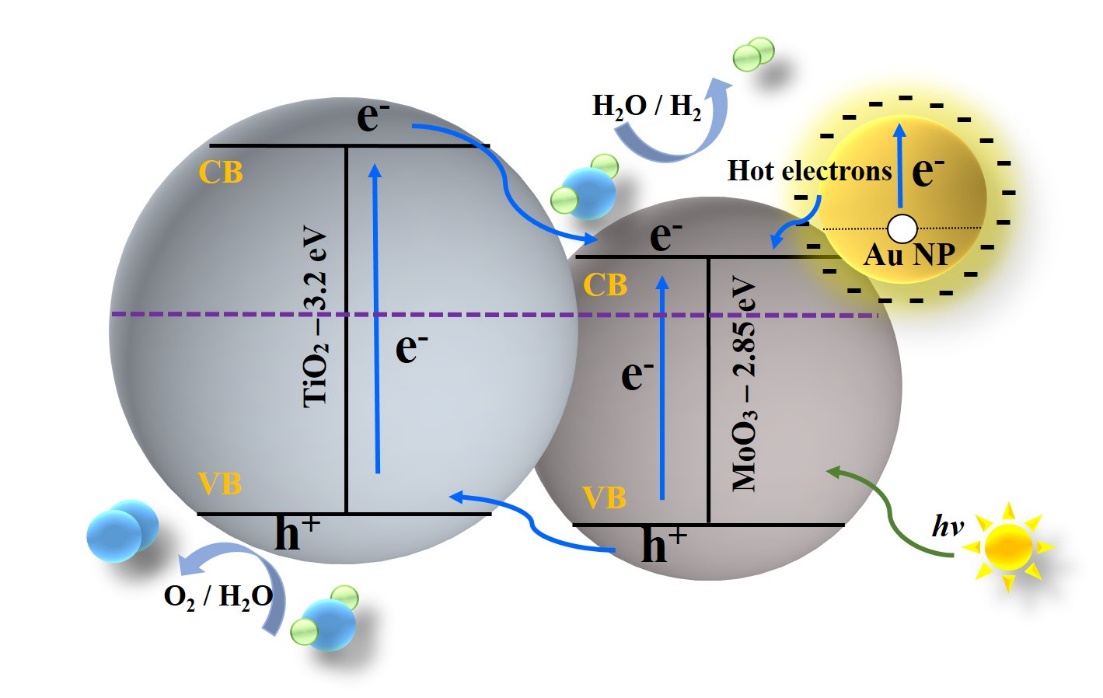


**Figure S4.** The enhancement mechanism of our Au/TNTs@MoO_3_ system.

The idea of forming semiconductor heterojunctions relies on the band energy alignment between the two semiconductors at the interface. For our work, the band alignment and bending at the heterojunctions created by two semiconductors with different band energy positions and Fermi levels. Once the junction is formed, electrons will flow from the semiconductor with the higher Fermi energy level (TNTs) to the semiconductor with the lower Fermi level (MoO_3_). This creates a built-in electric field at the interface with a potential difference between the two sides, shown in Figures 3, which improve the photo excited charges separation. The similar enhancement mechanism of TNTs-MoO_3_ system has also been mentioned lately in the application of photocatalysis.[^6-8^](#_ENREF_6)

Furthermore, to address this question more, we provide the illustration of the enhancement mechanism of our Au/TNTs@MoO_3_ system, beside the exsistence of the build-in heterojunction at TNTs@MoO_3_, hot electron generated from the decay of plasmon resonance in Au NPs, creating an additional improvement path in this system. These free hot electrons can be simultaneously transferred to the MoO_3_ conduction bands and conducted to the counter electrode to increase hydrogen generation.[^9-11^](#_ENREF_9)

**References**

1. Li, H.; Zhang, Q.; Yap, C. C. R.; Tay, B. K.; Edwin, T. H. T.; Olivier, A.; Baillargeat, D., From bulk to monolayer MoS_2_: evolution of Raman scattering. *Adv Funct Mater* **2012,** 22, 7, 1385-1390.

2. Plechinger, G.; Heydrich, S.; Eroms, J.; Weiss, D.; Schüller, C.; Korn, T., Raman spectroscopy of the interlayer shear mode in few-layer MoS_2_ flakes. *Appl Phys Lett* **2012,** 101, 10, 101906.

3. Luo, S.; Qi, X.; Ren, L.; Hao, G.; Fan, Y.; Liu, Y.; Han, W.; Zang, C.; Li, J.; Zhong, J., Photoresponse properties of large-area MoS_2_ atomic layer synthesized by vapor phase deposition. *J Appl Phys* **2014,** 116, 16, 164304.

4. Lee, C.; Yan, H.; Brus, L. E.; Heinz, T. F.; Hone, J.; Ryu, S., Anomalous lattice vibrations of single-and few-layer MoS_2_. *ACS nano* **2010,** 4, 5, 2695-2700.

5. Wieting, T.; Verble, J., Infrared and Raman Studies of Long-Wavelength Optical Phonons in Hexagonal MoS_2_. *Physical Review B* **1971,** 3, 12, 4286.

6. Papp, J.; Soled, S.; Dwight, K.; Wold, A., Surface acidity and photocatalytic activity of TiO_2_, WO_3_/TiO_2_, and MoO_3_/TiO_2_ photocatalysts. *Chem Mater* **1994,** 6, 4, 496-500.

7. Song, K. Y.; Park, M. K.; Kwon, Y. T.; Lee, H. W.; Chung, W. J.; Lee, W. I., Preparation of transparent particulate MoO_3_/TiO_2_ and WO_3_/TiO_2_ films and their photocatalytic properties. *Chem Mater* **2001,** 13, 7, 2349-2355.

8. Kong, F.; Huang, L.; Luo, L.; Chu, S.; Wang, Y.; Zou, Z., Synthesis and characterization of visible light driven mesoporous nano-photocatalyst MoO_3_/TiO_2_. *J Nanosci Nanotechno* **2012,** 12, 3, 1931-1937.

9. Pu, Y.-C.; Wang, G.; Chang, K.-D.; Ling, Y.; Lin, Y.-K.; Fitzmorris, B. C.; Liu, C.-M.; Lu, X.; Tong, Y.; Zhang, J. Z., Au nanostructure-decorated TiO_2_ nanowires exhibiting photoactivity across entire UV-visible region for photoelectrochemical water splitting. *Nano Lett* **2013,** 13, 8, 3817-3823.

10. Hung, W. H.; Lai, S. N.; Su, C. Y.; Yin, M.; Li, D.; Xue, X.; Tseng, C. M., Combined Au-plasmonic nanoparticles with mesoporous carbon material (CMK-3) for photocatalytic water splitting. *Appl Phys Lett* **2015,** 107, 7, 073904.

11. Li, Y.; Wei, X.; Zhu, B.; Wang, H.; Tang, Y.; Sum, T. C.; Chen, X., Hierarchically branched Fe_2_O_3_@TiO_2_ nanorod arrays for photoelectrochemical water splitting: facile synthesis and enhanced photoelectrochemical performance. *Nanoscale* **2016,** 8, 21, 11284-11290.
